# Robust genome and RNA editing via CRISPR nucleases in PiggyBac systems

**DOI:** 10.1016/j.bioactmat.2022.01.046

**Published:** 2022-02-07

**Authors:** Yuqian Jiang, Rachel Catherine Hoenisch, Yun Chang, Xiaoping Bao, Craig E. Cameron, Xiaojun Lance Lian

**Affiliations:** a Department of Biomedical Engineering, Pennsylvania State University, University Park, PA, 16802, USA; b Huck Institutes of the Life Sciences, Pennsylvania State University, University Park, PA, 16802, USA; c Department of Biology, Pennsylvania State University, University Park, PA, 16802, USA; d Davidson School of Chemical Engineering, Purdue University, West Lafayette, IN, 47907, USA; e Department of Microbiology and Immunology, University of North Carolina School of Medicine, Chapel Hill, NC, 27599, USA

**Keywords:** CRISPR-Cas9, Genome editing, Cas13d, RNA editing, PiggyBac transposon, Human pluripotent stem cells

## Abstract

CRISPR/Cas-mediated genome editing in human pluripotent stem cells (hPSCs) offers unprecedented opportunities for developing *in vitro* disease modeling, drug screening and cell-based therapies. To efficiently deliver the CRISPR components, here we developed two all-in-one vectors containing Cas9/gRNA and inducible Cas13d/gRNA cassettes for robust genome editing and RNA interference respectively. These vectors utilized the PiggyBac transposon system, which allows stable expression of CRISPR components in hPSCs. The Cas9 vector PB-CRISPR exhibited high efficiency (up to 99%) of inducing gene knockout in both protein-coding genes and long non-coding RNAs. The other inducible Cas13d vector achieved extremely high efficiency in RNA knockdown (98% knockdown for CD90) with optimized gRNA designs. Taken together, our PiggyBac CRISPR vectors can serve as powerful toolkits for studying gene functions in hPSCs.

## Highlights

• PB-CRISPR enables robust knockout of genes expressed in hPSCs.
• PB-CRISPR enables robust knockout of silent genes in hPSCs.
• PB-CRISPR enables robust knockout of lncRNAs in hPSCs.
• Inducible piggyBac Cas13d system enables robust RNA knockdown.

## Graphical abstract

**Figure:**
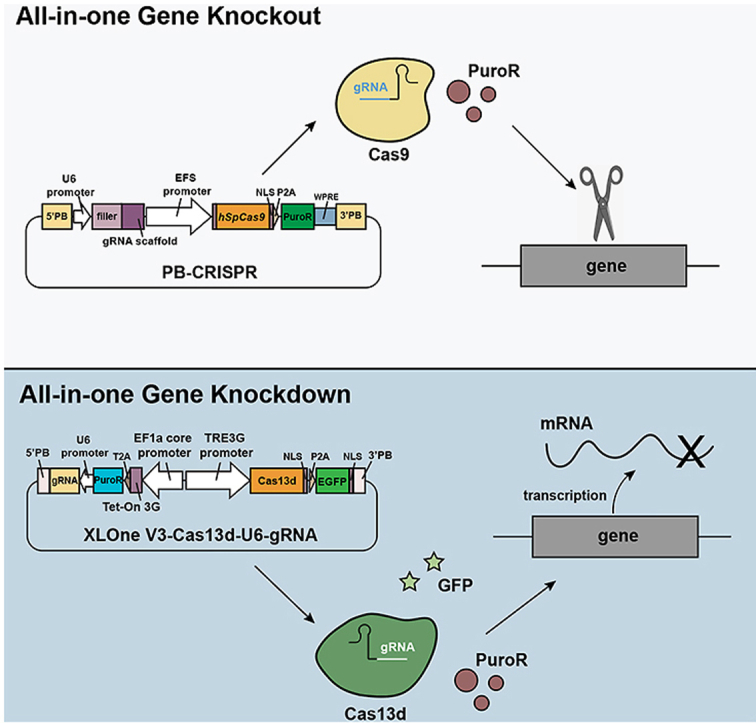

## 1 Introduction

The emergence of Clustered Regularly Interspaced Short Palindromic Repeat (CRISPR)/CRISPR-associated protein (Cas) technology opened a new era of gene editing in human cells [1,2]. Class 1 CRISPR systems usually rely on effector complexes made up of several Cas proteins, while Cas proteins in Class 2 systems could function as a single effector, which makes them more applicable for gene editing, such as Cas9 and Cas12 for DNA targeting [1,[3], [4], [5]] and Cas13 for RNA targeting [6,7]. Combination of CRISPR technology with human pluripotent stem cells (hPSCs) catalyzed a multitude of investigations on gene functions during human embryonic development via targeted gene interruptions. These studies have advanced our understanding of human development, accelerated various disease modeling and drug screening innovations [8,9].

**Fig. 1:**
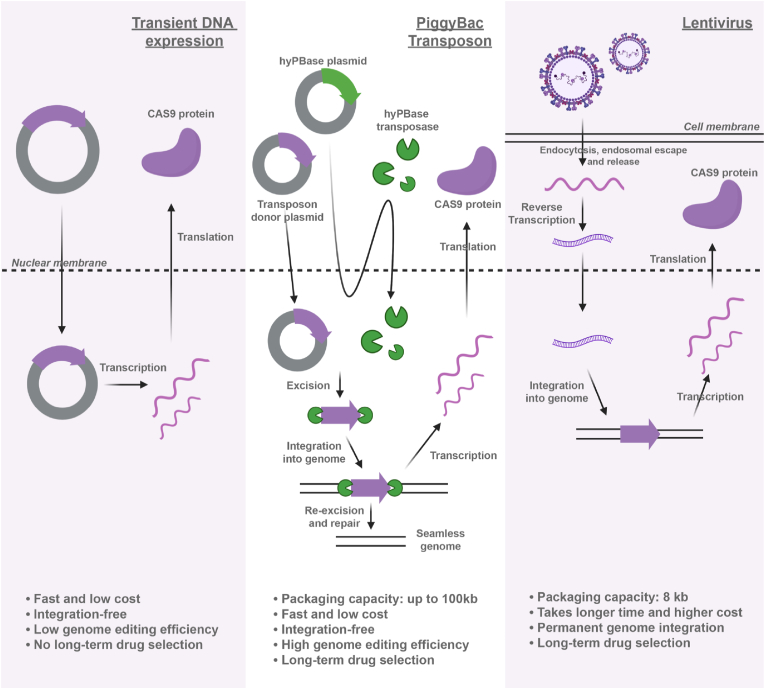
Comparison of different approaches for CRISPR delivery including transient DNA transfection, PiggyBac transposon system and lentiviral vectors.

CRISPR/Cas systems require efficient co-delivery of Cas proteins and gRNA into hPSCs for genome editing, which can be achieved via multiple approaches (Fig. 1). Transient expression of CRISPR components via plasmid transfection restricts its functioning window, greatly limiting the ultimate genome editing efficiency. Moreover, lack of a long-term drug resistance gene may result in the overgrowth of wildtype cells over modified cell populations, thus restricting the stable gene interruption. Lentiviral vectors for gene delivery enable continuous expression of CRISPR components. However, the 8 kb packaging capacity of lentivirus makes it less ideal for CRISPR cassettes [10]. In addition, it takes more time and cost to go through the lentivirus preparation, supernatant collection and virus concentration.

In contrast, PiggyBac-transposon system provides a versatile and efficient way for CRISPR-mediated genetic modifications. It has a much larger payload compared to lentiviral vectors, which is suitable for delivering large-size Cas proteins and gRNA [11]. In addition, its cut-and-paste mechanism allows for removal of insertions and generation of footprint-free modified cells [12,13]. Inclusion of a drug-selection gene or fluorescent reporters enables visualization or purification of the modified cells. Last but not least, PiggyBac plasmids are easy to clone and ready to use for gene delivery, greatly accelerating the speed of gene and RNA editing in hPSCs.

Here we generated all-in-one PiggyBac vectors delivering Cas9 and Cas13d-mediated cassettes for robust genome editing or RNA editing in hPSCs, which can serve as powerful tools for investigation of gene functions in hPSCs.

## 2 Results

### 2.1 PB-CRISPR enables robust knockout of protein-coding genes expressed in hPSCs

**Fig. 2:**
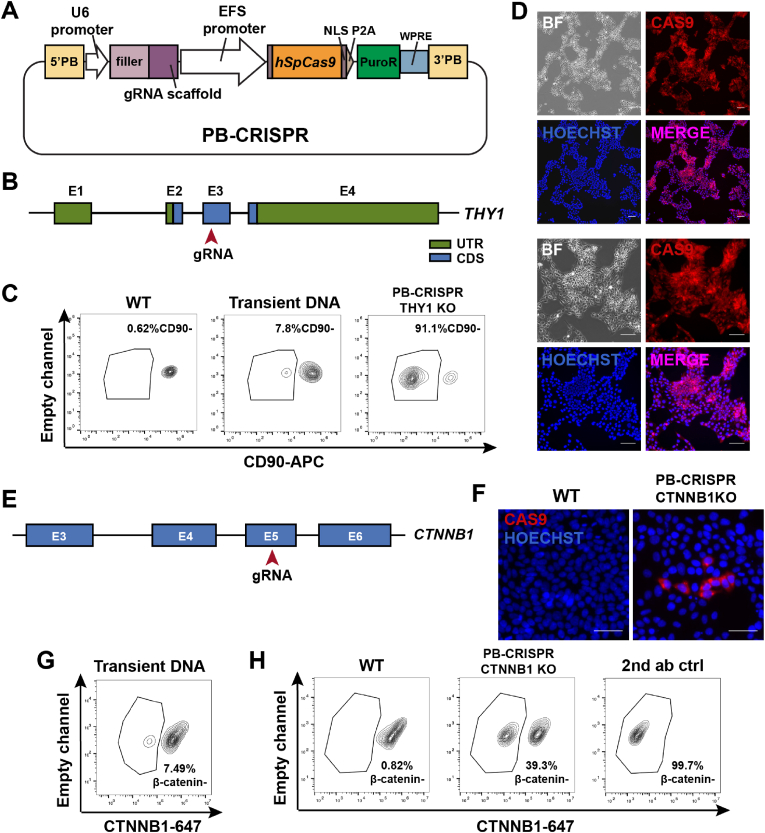
**PB-CRISPR enables robust knockout of protein-coding genes expressed in hPSCs.** A. Diagram of PB-CRIPSR plasmid design. B. Diagram of gRNA design to target *THY1* gene. UTR: untranslated region. CDS: coding sequence. C. Flow cytometry data of *THY1* knockout with either transient DNA delivery or PB-CRISPR insertion. D. Immunostaining images of IMR90C4 PB-CRISPR *THY1* KO cells against CAS9. Scale bar: 100 μm. E. Diagram of gRNA targeting *CTNNB1* gene. F. Immunostaining images of WT IMR90C4 cells or IMR90C4 PB-CRISPR *CTNNB1*KO cells against CAS9. Scale bar: 100 μm. G-H. Flow cytometry data of *CTNNB1* knockout with either transient DNA delivery or PB-CRISPR insertion.

We designed an all-in-one PiggyBac system PB-CRISPR to deliver both hSpCas9 and gRNA, along with a puromycin-resistant gene for drug selection (Fig. 2A). We first tested this system in protein-coding genes that are expressed in hPSCs (Fig. 2B–H and S1). *THY1* encodes CD90, which is a membrane glycoprotein expressed in hPSCs and has potential roles in cell adhesion and communication [14]. We picked a gRNA targeting the third exon of *THY1* gene and cloned it into our PB-CRISPR vector (Fig. 2B). Delivery of the PB-CRISPR plasmid alone resulted in transient expression of CRISPR cassettes in the cells and one week later, 7.8% CD90 negative cells were detected via flow cytometry (Fig. 2C). In comparison, co-delivery of PB-CRISPR and a plasmid expressing hyperactive transposase resulted in insertion of the Cas9-gRNA-Puro construct into genome. After 2 weeks of drug selection with puromycin, over 90% of hPSCs were CD90 negative (Figs. 2C and S1), which indicated that stable CRISPR expression via PB-CRISPR led to a much higher knockout efficiency than the transient DNA delivery. Stable expression of PB-CRISPR construct in drug-selected cells was confirmed by immunostaining against CAS9 (Fig. 2D).

Then we tested *CTNNB1,* another protein-coding gene which encodes for β-catenin that is constitutively expressed in hPSCs and functions as an important effector in Wnt signaling pathway. We designed a gRNA targeting the fifth exon that would cause long deletion in *CTNNB1* gene [15] (Fig. 2E). Stable expression of PB-CRISPR construct was confirmed by immunostaining against CAS9 (Fig. 2F). In contrast to the 7.49% knockout generated by transient plasmid transfection (Fig. 2G), stable expression of PB-CRISPR led to 39.3% cells with *CTNNB1* knockout after one week of puromycin selection (Fig. 2H). We also observed that extending drug selection for one more week did not further increase knockout efficiency, indicating that knockout efficiency by PB-CRISPR stable cell lines can reach maximum within one week of drug selection (Fig. S2A). Taken together these data demonstrated high efficiency of PB-CRISPR mediated protein-coding gene knockout can be achieved via generation of stable hPSC lines.

### 2.2 PB-CRISPR enables robust knockout of silent genes in hPSCs

**Fig. 3:**
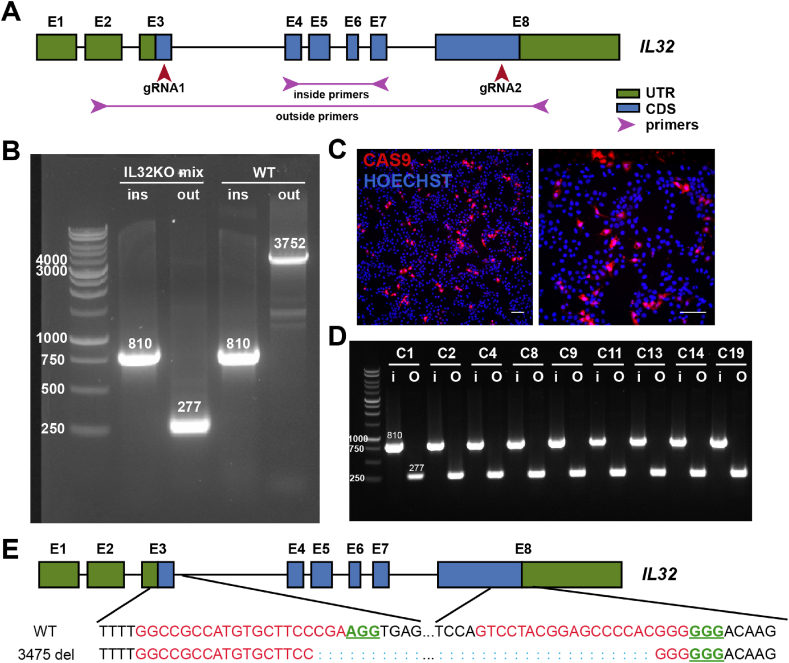
**PB-CRISPR enables robust knockout of protein-coding genes not expressed in hPSCs.** A. Diagram of gRNA design to target *IL32* gene and primer design for knockout genotyping. B. Gel images of PCR products for *IL32* knockout genotyping. C. Immunostaining images of IMR90C4 PB-CRISPR *IL32*KO cells against CAS9. Scale bar: 100 μm. D. Gel images of PCR products for *IL32* knockout genotyping with single cell derived colonies. E. Genotype of the truncated band with outside primers with *IL32* knockout IMR90C4 clone 1 cells.

We next tested if the system can be applied to genes that are not expressed in hPSCs, which takes more steps for genotyping. Here we chose gene *IL32*, which encodes for interleukin 32, a human proinflammatory cytokine. We designed two gRNAs that target the third and eighth exons in Coding DNA Sequence (CDS) region and two pairs of primers for genotyping (Fig. 3A). PCR with the genomic DNA from a cell mixture after nucleofection with PB-CRISPR-IL32KO and a PBase plasmid showed the generation of a short band with outside primers, which is 277 bp instead of the 3752 bp present in wildtype (WT) cells, indicating that at least one of the alleles has been modified due to Cas9 cutting (Fig. 3B). In addition, an 810 bp band with inside primers suggested that the other band is not fully deleted but could be modified partially in exon 3 or exon 8. After two months of drug selection with puromycin, we observed CAS9 expression retained in many cells (Fig. 3C) and thus derived single-cell clones from the mixture. PCR screening of all the single-cell clones showed a similar pattern as in mixed cells (Figs. 3D and S2B). Further genotyping revealed a 3475 bp deletion, which was located perfectly between two cutting sites of Cas9 (Fig. 3E).

### 2.3 PB-CRISPR enables robust knockout of lncRNAs in hPSCs

**Fig. 4:**
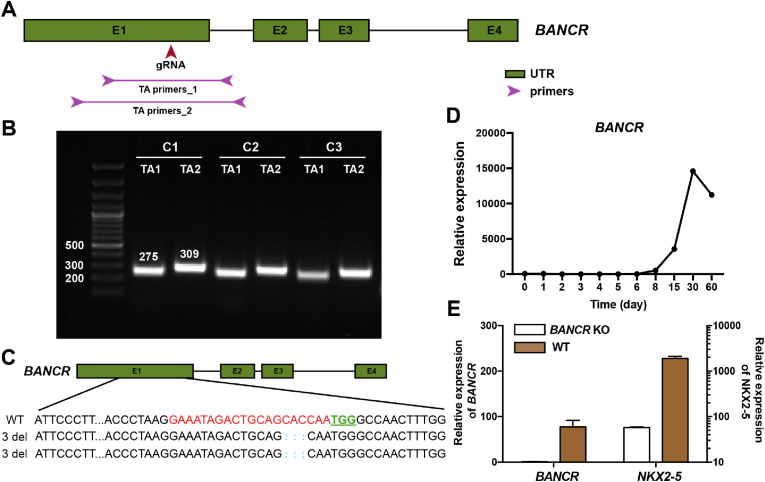
**PB-CRISPR enables robust knockout of non-protein-coding genes that are not expressed in hPSCs.** A. Diagram of gRNA design to target *BANCR* long non-coding RNA and primer design for knockout genotyping. B. Gel images of PCR products for *BANCR* knockout genotyping with single cell derived colonies. C. Genotype of the *BANCR* knockout *OCT4-GFP* H1 clone 1 cells. D. Dynamic *BANCR* expression during hPSC-CM differentiation. E. Relative expression of *BANCR* and *NKX2.5* in D30 CMs derived from WT or PB-CRISPR *BANCR* KO *OCT4-GFP* H1 cells.

We next applied PB-CRISPR to knock out long non-coding RNAs (lncRNA) that are not expressed in hPSCs. Here we picked gene *BANCR*, a lncRNA that was reported to be associated with cell proliferation, migration and invasion through the ERK signaling pathway [16]. A gRNA was designed to target the first exon of *BANCR* (Fig. 4A) and two pairs of primers were designed for genotyping. PCR reactions with three single cell-derived colonies showed no obvious truncated bands, indicating small insertion or deletions (Fig. 4B). Further TA cloning with clone 1 showed 3bp deletion in both alleles (Fig. 4C), which is consistent with the results from inDelphi, a reported platform for predicting CRISPR genome editing [17] (Fig. S2C). We previously found the dynamic expression pattern of *BANCR* during hPSC-cardiomyocyte (hPSC-CM) differentiation by bulk RNA sequencing, which was enriched on late stages starting from day 15 (Fig. 4D). With the identified *BANCR*-KO hPSC line, we observed negligible *BANCR* expression on day 30 of CM differentiation, compared with high *BANCR* expression in wildtype cells. Interestingly, we also observed significantly decreased expression in *NKX2.5*, which is associated with human heart development and formation of congenital heart defects (Figs. 4E and S2D). This indicated that *BANCR* may play a role in cardiac differentiation.

#### 2.3.1 Inducible PiggyBac Cas13d system enables robust RNA knockdown

**Fig. 5:**
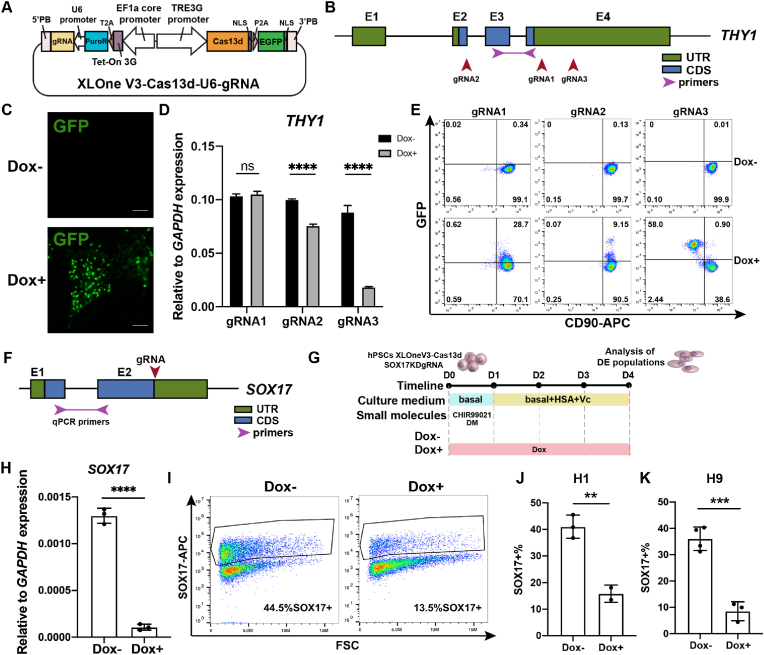
**Inducible Piggybac Cas13d system enables robust RNA editing.** A. Diagram of XLOne-Puro-Cas13d-eGFP-U6-gRNA plasmid design. B. Diagram of gRNA design to target *THY1* gene and primer design for qPCR experiments. C. Dox addition induced GFP expression in the nucleus of IMR90C4 XLOne-Puro-Cas13d-eGFP-U6-THY1gRNA cells. Scale bar: 100 μm. D. Relative expression of *THY1* RNA induced by different gRNA sequences. E. Flow cytometry of CD90 knockdown with different gRNA designs. F. Diagram of gRNA design to target *SOX17* gene and primer design for qPCR experiments. G. Diagram of definitive endoderm (DE) differentiation with H1 XLOne-Puro-Cas13d-eGFP-U6-SOX17gRNA cells with or without dox addition. Cells were treated with CHIR99021 and Dorsomorphin in basal medium on day 0 and then cultured in basal medium suppled with 0.05% HSA and 200 μg/mL ascorbic acid for the next three days. H. Relative expression of *SOX1*7 RNA on D4 with or without dox. I. Flow cytometry stained against SOX17 on day 4 with H1 XLOne-Puro-Cas13d-eGFP-U6-SOX17gRNA cells with or without dox addition. J-K. Quantification of Cas13d-mediated SOX17 knockdown efficiency in differentiated DE cells from H1 cell line (J) or in H9 cell line (K).

Besides genome editing, we also constructed an inducible all-in-one PiggyBac system (XLOne-Puro-Cas13d-eGFP-U6-gRNA) for RNA editing, which contains PiggyBac inverted terminal repeats that flank multiple gene elements that are driven by three promoters. The first promoter is a EF1a core promoter, which controls the expression of Tet-On 3G transactivator protein and puromycin-resistant gene, followed by a gRNA sequence driven by the U6 promoter. The third promoter is a TRE3G promoter controlling the expression of Cas13d and eGFP fluorescent reporter (Fig. 5A). We first applied this system to interrupt the gene *THY1* due to its surface expression in hPSC stage. Three gRNAs were designed [18] and cloned to test the editing efficiency (Fig. 5B). IMR90C4 iPSCs were incorporated with XLOne-Puro-Cas13d-eGFP-U6-THY1gRNA plasmid and selected by puromycin for about two weeks. Addition of doxycycline (dox) induced eGFP expression in iPSCs, indicating successful construct design (Fig. 5C). After four days of dox treatment, we collected cells to test the *THY1* expression in the RNA level via qPCR and in the protein level via flow cytometry (Fig. 5D and E). qPCR data revealed distinct performance of three gRNAs in *THY1* KD (Fig. 5D), where gRNA2 and gRNA3 led to significant *THY1* mRNA decline, while gRNA1 showed no effects on *THY1* KD. Our flow cytometry of CD90 expression results demonstrated that gRNA3 could almost deplete THY1 protein expression, achieving 98% knockdown efficiency (Fig. 5E).

Next we tested our Cas13d knockdown system on *SOX17*, which is a marker gene for definitive endoderm [19]. Cas13d gRNA was designed to target the 3′UTR region (Fig. 5F). H1 hESC line integrated with XLOne-Puro-Cas13d-eGFP-U6-SOX17gRNA was used for DE differentiation using our small-molecule GiBi protocol [19] with or without dox treatment (Fig. 5G). Specifically, cells were treated with CHIR99021 and Dorsomorphin on day 0 and then cultured in a basal medium supplemented with human serum albumin and ascorbic acid for the next three days. Daily images tracked the morphology change with or without dox treatment (Fig. S3A). Dox addition led to lower cell confluency on day 1 and 2 of DE differentiation. In addition, day 4 cells under dox treatment failed to generate dense clusters and some cells still exhibited stem cell morphology with large nuclei. qPCR data of day 4 cells revealed that dox treatment led to significant decrease of *SOX1*7 mRNA expression (Fig. 5H). A significant decrease of SOX17 positive cell percentage from an average 40% to 15% was also observed after dox addition (Fig. 5I and J). The construct exhibited similar function for SOX17 knockdown in another H9 hESC line (Fig. 5K). To rule out the possibility that SOX17 knockdown arose from dox toxicity, we also tested the DE efficiency using the WT H1 cells with or without the presence of dox and found that dox addition did not impair the SOX17 expression in day 4 DE cells (Fig. S3B). All these data demonstrated our Cas13d system can induce robust RNA knockdown.

## 3 Discussion

In this study, we generated two all-in-one PiggyBac vectors containing Cas9/gRNA cassettes for robust genome editing or inducible Cas13d/gRNA for RNA knockdown, along with drug resistance genes allowing the generation of stable hPSC lines for gene studies. The Cas9/gRNA vector PB-CRISPR showed high efficiency of inducing gene deletions in both protein-coding genes and long non-coding RNAs, and the other inducible Cas13d/gRNA plasmid (XLOne-Puro-Cas13d-eGFP-U6-gRNA) also performed well in RNA knockdown with optimized gRNA designs.

Multiple elegant strategies have been reported to deliver CRISPR elements in previous studies [12,[20], [21], [22]]. The Barrett group established hPSC lines with AAVS1-harboring dox-inducible fluorescent-labeled (EGFP) expression of dCas9-KRAB or dCas9-VPR [20]. To enable gene interference (CRISPRi) or gene activation (CRISPRa), another PiggyBac-mediated vector expressing multiple gRNAs labeled by mRFP and blasticidin (bsd) resistance design was further delivered. These dual fluorescent CRISPRi/a hPSC lines facilitated functional dissection of multiple genes and pathways for studies of development and disease. However, related applications are merely limited to their established hPSC lines. In contrast, our all-in-one vectors for efficient gene or RNA interference can be easily adapted in other cell types, although hPSCs were used as examples in our work, by avoiding the time-consuming and much less efficient targeted knockin process and separate steps in delivering Cas protein and gRNA cassettes. The Liu group developed a PB transposon-based CRISPRa system with co-delivery of multiple gRNAs along with dCas9-VP64 and bsd resistance gene, which was demonstrated for stable and simultaneous activation of multiple transcription factors and long non-coding RNAs to drive the differentiation from iPSCs to neural cell fates [21]. Here our all-in-one vectors presented similar designs to contribute to the applications for gene knockout or RNA interference that their work didn't cover. We can also further modify our design by adding multiple gRNA expressing cassettes as they did to realize multi-gene-targeting for future applications. The Pu group and the Calabrese group both reported the development of PB-mediated inducible expression of Cas9 along with drug resistance gene for gene editing [12,22]. But they both require additional gRNA delivery in the form of oligos or as a second PB vector. Other studies utilizing transient delivery of Cas9 nucleases instead of with PB systems requires more genotyping to isolate and identify modified single clones [[23], [24], [25], [26]].

Collateral RNA cleavage (non-targeted ssRNA cleavage) has been shown to occur when the Cas13-crRNA complex is hybridized with a target RNA [6,7,[27], [28], [29]], which is utilized to develop various biosensors for sensitive nucleic acid detection [30]. This non-targeted RNA cleavage by Cas13d has been shown to cause toxicity and cell death in bacteria [31], but is less observed or reported in hPSCs or other eukaryotic cells [[32], [33], [34], [35]], which makes it less of a concern for RNA knockdown in hPSCs. In our study, we also didn't see severe cell death in Cas13d-induced gene knockdown. A recent study discovered a new Cas7-11 protein that also presented robust RNA targeting as well as negligible collateral RNA cleavage [36]. Although it has a much larger size than Cas13d, Cas7-11 could be the next powerful effector to be cloned into our all-in-one PiggyBac systems for targeted RNA interference.

## 4 Experimental procedures

### 4.1 hPSC maintenance and nucleofection

hESCs (H1, H9, *OCT4-GFP* H1 [37]) and IMR90C4 iPSC line [38] were obtained from WiCell Research Institute. Undifferentiated hPSCs were maintained on iMatrix-511 SILK (Iwai North America) coated plates in mTeSR1 medium (Stemcell Technologies). When cells were 80–90% confluent, medium was aspirated and 1 mL Accutase (Innovative Cell Technologies) was added to each well. Cells were incubated at 37 °C, 5% CO_2_ for 10 min. Dissociated cells were transferred into excess DMEM at a 1:2 (vol/vol) ratio and centrifuged at 1000 rpm for 4 min. Cell pellet was resuspended in mTeSR1 with 5 μM Y-27632 and 1.5 μL iMatrix-511 SILK per mL media and 10,000–20,000 cells/cm^2^ were seeded into wells. Incubated at 37 °C, 5% CO_2_, hPSCs were routinely tested for mycoplasma and all the cells were negative for mycoplasma contamination.

For hPSC transfection, around 2 million cells were dissociated and centrifuged as described above. Pelleted cells were resuspended with 100 μL DNA-containing DPBS and were nucleofected with Lonza 4D-nucleofector using CA137 code. Cells were then recovered in 1 mL pre-warmed mTeSR1 medium supplemented with 5 μM Y-27632 at 37 °C, 5% CO_2_ for 10 min and replated to one well of a six-well plate in a total 2 mL medium.

### 4.2 Cardiomyocyte differentiation (small-molecule GiWi protocol)

Differentiation started when cells were at least 80% cell confluency. On day 0, cells were treated with CHIR99021 (CH) of optimized concentration in RPMI for 24 h, followed by media change to RPMI plus B-27 without insulin supplement for 48 h. On day 3 of differentiation, cells were treated with 2 μM Wnt-C59 in RPMI plus B-27 without insulin supplement for 2 days. From day 5, cells were cultured in RPMI plus B-27 supplement, with media change every three days.

### 4.3 DE differentiation

DE differentiation was initiated when the hPSCs reached 70–80% confluency. H1 hESCs were treated with 3 μM CH (Cayman Chemical) and 1 μM Dorsomorphin (DM) (Sigma Aldrich) for 24 h. Cells were then cultured for another three days in a basal medium containing 0.05% HSA and 200 μg/mL ascorbic acid. H9 hESCs were treated with 3 μM CH and 1 μM DM for 24 h. Cells were then cultured for another two days in a basal medium with B-27 supplement.

### 4.4 PCR for genotyping

Genomic DNA was extracted from cells with Quick-DNA Miniprep Plus Kit (Zymo research). DNA concentration was measured with Nanodrop spectrophotometer. PCR reactions were set up with primers, template DNA and GoTaq green master mix (Promega). PCR products were loaded and imaged in 1% agarose gel and imaged with ChemiDoc Touch Imaging System (Biorad).

### 4.5 TOPO TA cloning for genotyping

PCR products with genomic DNA and TA primers were purified with the Zymo DNA clean and concentrate kit. A TOPO cloning reaction was set up following the instruction (ThermoFisher Scientific). Incubate for 10 min at room temperature and place the reaction on ice. Transform 1 μL reaction to E.coli and incubate overnight. Pick at least 8 E.coli colonies for Sanger sequencing with primers targeting T7 or T3 promoter.

### 4.6 qPCR

Cells were lysed with TRI-Reagent for 1 min and RNA was extracted with Direct-zol RNA Miniprep Plus kit (Zymo research). cDNA was synthesized with Maxima First Strand cDNA Synthesis Kit (Life Technologies). Quantitative PCR reactions were performed using the SYBR Green PCR master mix (Life technologies) and run on a CFX Connect real-time qPCR machine (Bio-Rad). *GAPDH* was used as the house keeping gene for reference. Data were analyzed with the ΔC_T_ method unless otherwise indicated. Primers are listed in Table S1.

### 4.7 Immunostaining

Cells were fixed with 4% formaldehyde at room temperature for 15 min and then blocked in DPBS with 0.4% Triton X-100 and 5% non-fat dry milk for 1 h. After that, cells were sequentially stained with primary and secondary antibodies (Table S2). Nuclei were stained with Hoechst 33,342 (ThermoFisher). Images were captured using a Nikon Ti Eclipse epifluorescence microscope. Images were processed using ImageJ software.

### 4.8 Flow cytometry

Cells were dissociated with Accutase for 10 min. For flow cytometry with live cells, cells were resuspended in FlowBuffer-1 (DPBS with 0.5% BSA). For flow cytometry analysis using fixed cells, 1% formaldehyde in DPBS was used to fix cells for 30 min. After that, cells were stained with primary and secondary antibodies (Table S2) in FlowBuffer-2 (DPBS with 0.5% BSA and 0.1% Triton X-100). Data were collected on a BD Accuri C6 plus flow cytometer and were processed in Flowjo software.

### 4.9 Statistical analysis

Quantification of flow cytometry data is shown as mean ± s.d. unless otherwise indicated. Unpaired two-tailed student's t-test was used for comparison between two groups. P values ≥ 0.05 were considered not significant; *P < 0.05, **P < 0.01, ***P < 0.001, ****P < 0.0001 were considered significant.

## CRediT authorship contribution statement

**Yuqian Jiang:** designed the experiments, performed the experiments, wrote. **Rachel Catherine Hoenisch:** assisted with the plasmid cloning. **Yun Chang:** performed the experiments, assisted with the plasmid cloning. **Xiaoping Bao:** designed the experiments. **Craig E. Cameron:** revised the manuscript. **Xiaojun Lance Lian:** designed the experiments, performed the experiments, revised the manuscript, approved the final draft of the manuscript.

## Declaration of competing interest

The authors declare no competing interests. Human embryonic stem cell work was approved by the Embryonic Stem Cell Oversight Committee at the Pennsylvania State University.
