# Supplementary material for: Robust genome and RNA editing via CRISPR nucleases in PiggyBac systems
Source: Bioact Mater. 2022 Feb 7;14:313–20. doi: 10.1016/j.bioactmat.2022.01.046 (PMC8964983; doi:10.1016/j.bioactmat.2022.01.046)
Supplement: Multimedia component 1 [file mmc1.pdf]

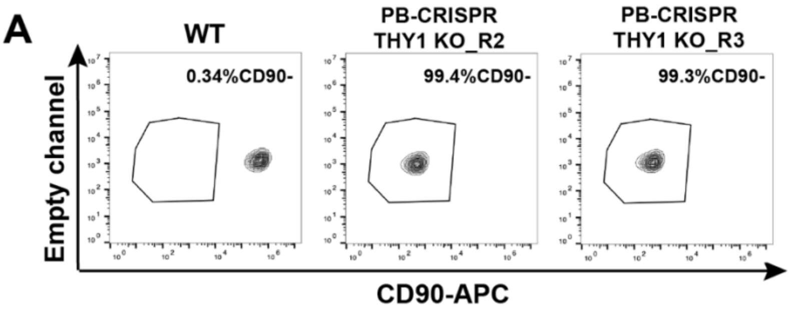

Figure S1. A. (Related to Fig. 2C) Two more biological replicates (repeat 2 and repeat 3) of flow cytometry data of *THY1* knockout with PB-CRISPR.

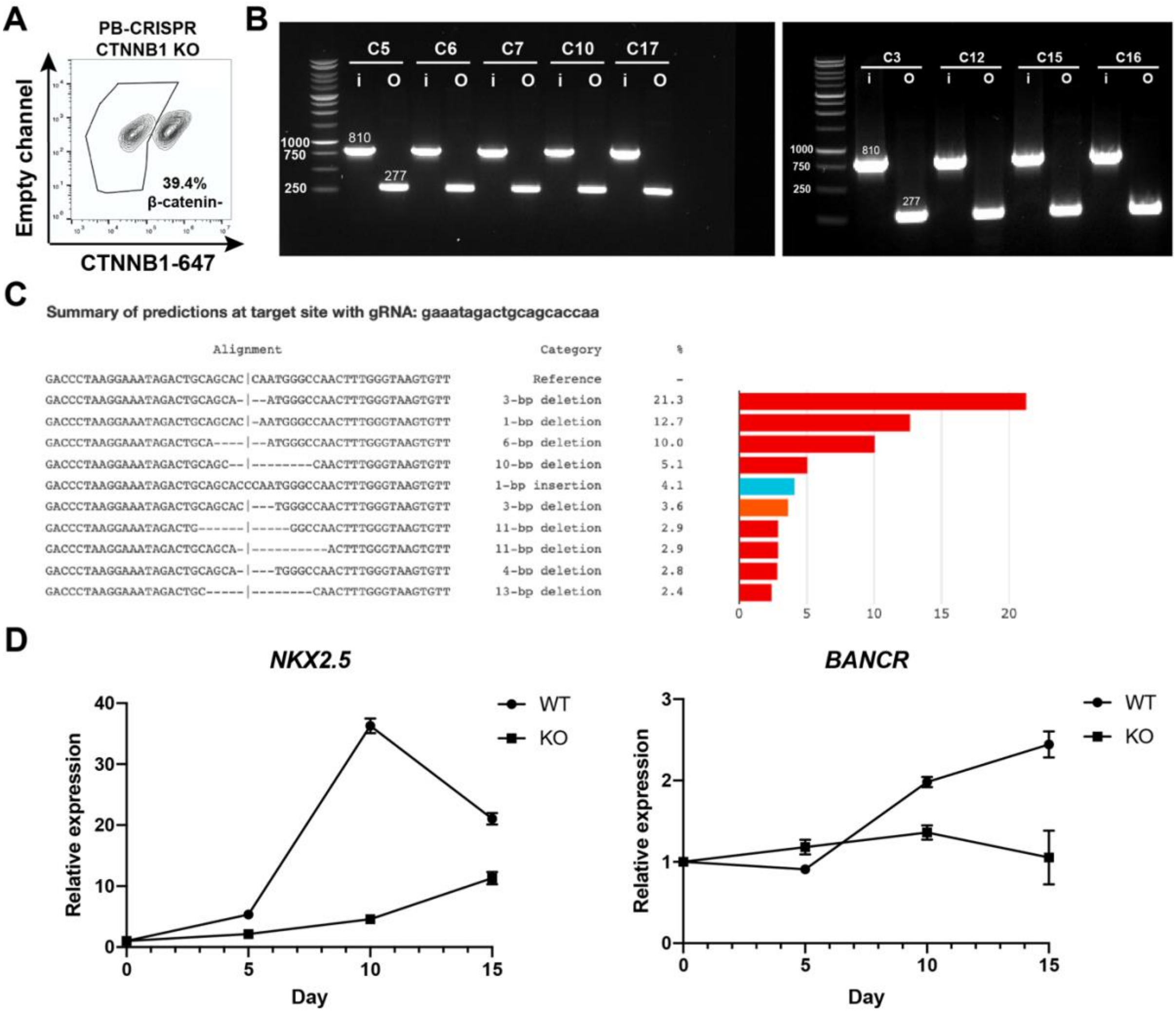

Figure S2. A. Flow cytometry against β-catenin with IMR90C4 PB-CRISPR CTNNB1KO cells after two weeks of Puromycin selection. B. Gel images of PCR products for IL32 knockout genotyping with more single cell derived colonies. C. Indelphi (https://indelphi.giffordlab.mit.edu) predicted that 3bp deletion is the most likely mutation with indicated gRNA design. D. Dynamic RNA expression of *NKX2.5* and *BANCR* on day 0, 5, 10, and 15 of CM differentiation with WT or PB-CRISPR *BANCR*KO *OCT4-GFP* H1 cells. ΔΔCt was performed.

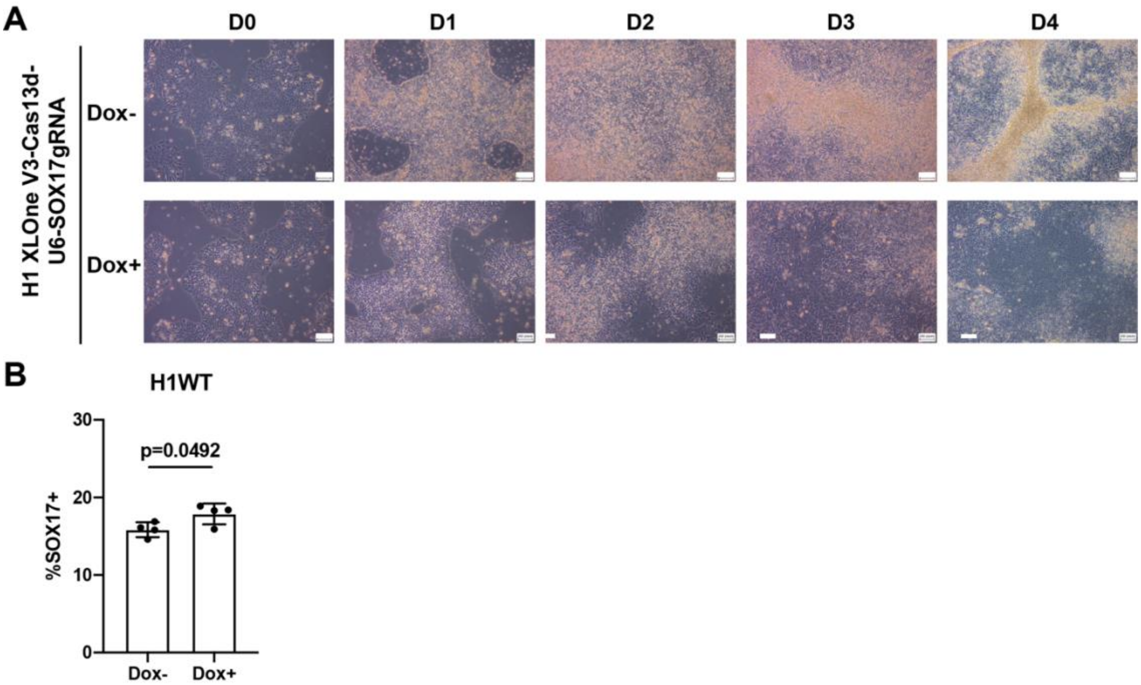

Figure S3. A. Daily images with XLOne-Puro-Cas13d-eGFP-U6-SOX17gRNA H1 cells during DE differentiation with or without Dox treatment. Scale bar: 130 µm. B. H1 WT cells were induced for DE differentiation with or without the presence of 5ug/mL dox. Cells were harvested for flow cytometry stained against SOX17 on day 4.

Table S1. gRNA and Primers

THY1KO gRNA	CATGGCGGCAGTCCAGACGA
CTNNB1KO gRNA	GAAACAGCTCGTTGTACCGC
IL32KO gRNA1	GGCCGCCATGTGCTTCCCGA
IL32KO gRNA2	GTCCTACGGAGCCCCACGGG
IL32KO inside primer_forward	AGAAGCTGAAGGCCCGAAT
IL32KO inside primer_reverse	TCCAGGTAGCCCTCTTTGAA
IL32KO outside primer_forward	ATTTGTGCCAGGAAGACTGC
IL32KO outside primer_reverse	GGGCAAAGGTGGTGTCAGTA
BANCRKO gRNA	GAAATAGACTGCAGCACCAA
BANCRKO TA primers_1_forward	TGTAGGGTCTGGATTGGGAC
BANCRKO TA primers_1_reverse	TTGCGTCTCAAACCCAAGTC
BANCRKO TA primers_2_forward	TGTGTGAGATCCAAGAACCTTC
BANCRKO TA primers_2_reverse	ACCTTCCTAAGTTGCGTCTCA
BANCR_qPCR_forward	GATTGGGACCCTTTTCTGGT
BANCR_qPCR_reverse	TTCCTTAGGGTCAGGGGTCT
NKX2.5_qPCR_forward	AGAGCCGAAAAGAAAGCCTG
NKX2.5_qPCR_reverse	CCGCACAGTAATGGTAAGGG
THY1KD gRNA1	AAAAAGTACAAAAAGACAGCCAG
THY1KD gRNA2	CAAGACTGTTAGCAGGAGAGCGA
THY1KD gRNA3	TAAACCAGACAGAAGCAGCTCTG
THY1_qPCR_forward	GAAGGTCCTCTACTTATCCGCC
THY1_qPCR_reverse	TGATGCCCTCACACTTGACCAG
SOX17KD gRNA	ACCATAAATTATATGCCAACACA
SOX17_qPCR_forward	GGCGCAGCAGAATCCAGA
SOX17_qPCR_reverse	CCACGACTTGCCCAGCAT

Table S2. Antibodies

**Name**	**Catalog number**	**Dilution**
CAS9	Biolegend, #844301, mouse IgG1	1:100
β-Catenin	BD, #610153, mouse IgG	1:200
CD90-APC	Biolegend, #328113, mouse IgG1	1:60
SOX17-APC	R&D systems, #IC1924A, goat IgG	1:50
Goat anti-mouse IgG (H+L), Alexa Fluor 647	Thermo Fisher Scientific Invitrogen #A-21235	1:1000

Table S3. Plasmids

Name	Catalog number
PB-CRISPR	Addgene, #160047
XLOne-Puro Cas13d-eGFP U6-BbsI	Addgene, #155184
XLOne-Puro Cas13d-eGFP U6-SOX17gRNA1	Addgene, #155187
